# Why Vaccinate Against COVID-19? A Population-Based Survey in Switzerland

**DOI:** 10.3389/ijph.2022.1604226

**Published:** 2022-03-23

**Authors:** Marta Fadda, Anne Linda Camerini, Maddalena Fiordelli, Laurie Corna, Sara Levati, Rebecca Amati, Giovanni Piumatti, Luca Crivelli, L. Suzanne Suggs, Emiliano Albanese

**Affiliations:** ^1^ Institute of Public Health, University of Italian Switzerland, Lugano, Switzerland; ^2^ Department of Business Economics, Health and Social Care, University of Applied Sciences and Arts of Southern Switzerland (SUPSI), Manno, Switzerland; ^3^ Unit of Development and Research in Medical Education, Université de Genève, Geneva, Switzerland

**Keywords:** vaccine hesitancy, pandemic, vaccination, Switzerland, COVID-19

## Abstract

**Objectives:** This study examined factors associated with COVID-19 vaccination intention at the very beginning of the vaccination campaign in a representative sample of the population in southern Switzerland.

**Methods:** In March 2021, we measured vaccination intention, beliefs, attitudes, and trust in a sample of the Corona Immunitas Ticino study.

**Results:** Of the 2681 participants, 1933 completed the questionnaire (response rate = 72%; 55% female; mean_age_ = 41, SD = 24, range_age_ = 5–91). Overall, 68% reported an intention to get vaccinated. Vaccination intention was higher in social/healthcare workers, and increased with age, trust in public health institutions, and confidence in the vaccine efficacy. Prior infection of a family member, predilection for waiting for more evidence on the safety and efficacy of the vaccine, and for alternative protective means were negatively associated with intention.

**Conclusion:** In view of needs of COVID-19 vaccine boosters and of suboptimal vaccination coverage, our results have relevant public health implications and suggest that communication about vaccine safety and efficacy, and aims of vaccination programs, should be bi-directional, proportionate, and tailored to the concerns, expectations, and beliefs of different population subgroups.

## Introduction

**FIGURE 1:**
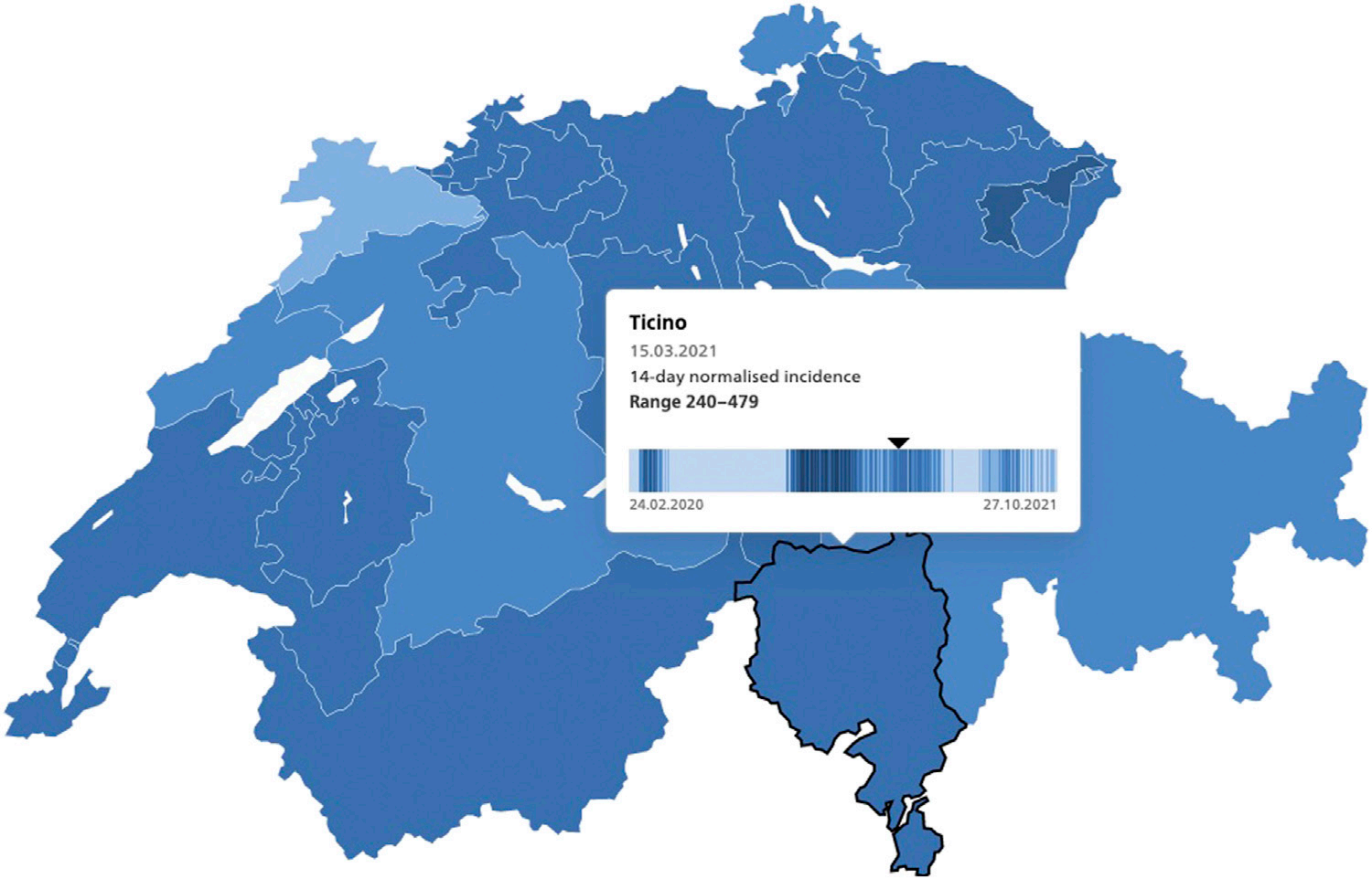
14-day normalized incidence of COVID-19 laboratory-confirmed cases, Switzerland, Ticino, 15.3.2021, source: https://www.covid19.admin.ch/en/epidemiologic/case.

Following the approval of the first COVID-19 vaccines by Swissmedic in December 2020, vaccination campaigns began across Switzerland (1). In Ticino, the Italian speaking canton that borders the heavily affected regions in northern Italy, vaccinations began in January 2021 prioritizing older adults and frontline healthcare and social workers (2). In the first quarter of 2021, Switzerland administered, free of charge at the point of delivery (3), the mRNA vaccines from Pfizer-BioNTech and Moderna (4). In mid-March 2021, when the present study was carried out, the 14-day incidence of confirmed cases per 100,000 inhabitants at the national level was 194. In Ticino, this number ranged from 240 to 479 cases (see Figure 1) (5).

The availability of COVID-19 vaccines is crucial for protection and to reduce the risk of severe disease through adequate immunization coverage. However, availability alone is not sufficient to achieve these aims (6–10). Vaccination decisions are influenced by several interacting drivers, including emotional, cultural, social, religious, logistical, political, and cognitive factors (11–13). The COVID-19 pandemic entails additional and unique challenges for public confidence in vaccines (14, 15). The development of vaccines was exceptionally fast, data on both safety and efficacy is, to date, short-term. Its use was initially authorized under emergency use terms (EUA) (16), and the composition, functioning and technology of most COVID-19 vaccines are relatively novel (17). Furthermore, concerns about the Oxford-AstraZeneca and Johnson and Johnson vaccine in other countries triggered safety concerns and confusion in the public (14, 18, 19). Another considerable challenge is posed by the *infodemic* associated with the pandemic and the unprecedented spread of misinformation, which does not spare vaccines (20–22). These factors contribute to vaccine hesitancy, defined as a “delay in acceptance or refusal of vaccination despite availability of (safe and efficacious) vaccines” (11) (p. 4163).

Global COVID-19 vaccination acceptance was ≥70%, but with marked geographic variations (23). Intention to get vaccinated ranged from 28% in Congo to 93% in China during the first year of the pandemic (24, 25). Lower levels of education and working in the healthcare sector were associated with lower intention (26). Some personality traits and attitudes, having received an influenza vaccination in the last year, and perceived threat to physical health were all associated with greater intention (23, 27).

Serological testing has become more common and accessible through large serosurveys used to measure the extent of the COVID-19 infection in populations. However, little is known about the potential modulating effect of known prior infection on vaccination intention. Callaghan et al. (28) found that past infection with COVID-19 was negatively correlated with vaccine uptake. Exposure to the virus conceivably confers protection against re-infection and/or some level of functional immunity against secondary severe COVID-19 disease (29). Therefore, knowledge of immune memory from primary infection(s) may lead to a lower intent to vaccinate against COVID-19. Moreover, evidence on vaccination intention in older adults, who are more prone to develop severe symptoms, and children, for whom vaccines were approved in December 2021, is extremely sparse, but very important (30). Evidence on vaccination intention in teenagers and children, who are often asymptomatic carriers (31), is crucial to inform public health decisions, and may contribute to attaining herd immunity (32, 33).

### Aim and Research Questions

The aim of this study was to measure the intention to get vaccinated against COVID-19, to identify the attitudes and beliefs associated with COVID-19 vaccination and to assess their role, together with socio-demographic factors and known prior infection of the self or a family member, in predicting intention during the early phase of the vaccination campaign, in a representative sample of the population of southern Switzerland (Canton Ticino). In 2019 (34), there were 8.6 million inhabitants in Switzerland, of which 20% aged 0–19, 61% aged 20–64, and 19% aged 65 years or older. Canton Ticino had approximately 350′000 inhabitants in 2019, with 18% aged 0–19, 59% aged 20–64, and 23% aged 65 years or older. Foreign nationals constituted 25% of the population at the national level and 28% in Ticino.

The study addresses the following three research questions (RQ):

RQ1: How many individuals are likely or very likely to decide to get vaccinated against COVID-19 among the general population residing in Ticino, stratified by age groups?
RQ2: What are the most common COVID-19 vaccination-related beliefs among the general population residing in Ticino?
RQ3: What is the role of general and COVID-19 vaccination-related beliefs, attitudes, socio-demographic factors, and known prior infection in predicting COVID-19 vaccination intention?

## Methods

### Study Design, Recruitment, and Procedures

The data for this study come from the prospective cohort, population-based Corona Immunitas Ticino study aiming to assess seroprevalence, and the impact of the COVID-19 pandemic in Ticino.

Between July and September 2020, 13′226 invitation letters were sent to a randomly selected, age stratified sample of residents extracted from the residential registry in Ticino: in July to adults aged 20–64 (*n* = 4000), and in September to parents of children aged 5–13 (*n* = 2170), teenagers aged 14–19 (*n* = 2094), and older adults aged 65+ (*n* = 4962). Diplomats, people under guardianship/asylum, those with a short-term residence permit, older adults living in long-term facilities were not sampled. Italian speaking participants (or their legal representative) who provided informed consent were enrolled in the digital cohort study upon completion of an online registration form and a baseline questionnaire. We implemented repeated questionnaires in REDCap (35, 36). For children aged 5–13, parents completed the questionnaires with reference to the participating child. Older adults with limited Internet access and digital skills were interviewed by a dedicated interviewer using computer assisted telephone interviewing.

We administered additional ad-hoc surveys to participants in specific phases of the project, including 1) a sero-specific questionnaire at the time of blood collection and 2) a vaccination questionnaire collecting data for the present study. The vaccination questionnaire was sent in March 2021 to participants in the digital cohort (*n* = 2681). They received a reminder after 2 weeks from the release of the questionnaire. The study was approved by the Cantonal Ethics Committee (2020-01514).

### Measures

#### COVID-19 Vaccination Intention

We measured vaccination intention with the item “Once the coronavirus vaccine is available to you (your child), how likely is it that you will decide to get (your child) vaccinated?” on a 5-point Likert scale ranging from 1 “very unlikely” to 5 “very likely”.

#### COVID-19 Vaccines and Vaccination Beliefs

We used 22 items from previously validated scales (37–39) and previous studies (14) to measure general and COVID-19-related vaccine and vaccination beliefs. We explored perceived efficacy, perceived safety, and preference for natural immunity (see Supplementary Table S1 in Supplement for a complete list of all items). We scaled responses using a 5-point Likert scale ranging from 1 “strongly disagree” to 5 “strongly agree”.

#### Attitude and Trust Towards Vaccination

We measured attitude and trust towards vaccination with a 6-item scale, the Vaccination Acceptance Index (VAI) (40), adapted to the Swiss context. The scale includes one item measuring vaccination confidence, and five items on trust, each using a 5-point Likert scale ranging from 1 “strongly disagree” to 5 “strongly agree” (see Supplementary Table S1 in Supplement). Following the proposed procedure, we converted all items to a scale from 0–100 with higher scores indicating higher acceptance levels (41). The scale showed high internal consistency (Cronbach’s alpha = 0.902).

#### Prior Infection

To assess prior COVID-19 infection, we carried out SARS-CoV-2-IgG antibody testing with a previously validated Luminex assay on sera obtained from peripheral venous blood (42), combined with self-reported positive PCR or serology test results from the baseline and follow-up questionnaires. We also assessed whether participants were aware of a positive PCR or serology test result of a family member. We created two dummy variables, for the participant and the family member, respectively, with 1 indicating “at least one positive test result, either lab-confirmed or reported” and 0 indicating “negative or no test result reported”.

#### Socio-Demographics

In the baseline questionnaire, we assessed gender, age, highest educational attainment (for participants <20 years, the highest educational attainment of their parents), nationality, number of household members, perceived financial situation and, for participants ≥20 years who indicated to be (self-)employed, whether they worked in the social or healthcare sector, and/or had direct contact with children or youth.

### Data Analysis

We used SPSS^©^ v.24 for all statistical analysis. We excluded participants who reported to have already received COVID-19 vaccination (*n* = 148) and participants who did not answer to at least 50% of the vaccination questionnaire (*n* = 600). For the remaining analytic sample (*n* = 1933), we imputed missing values on vaccination intention, beliefs, attitudes, and trust items for 223 (21.5%) respondents using an Expectation-Maximization algorithm. We conducted χ^*2*^- and independent samples *t*-tests to assess differences in socio-demographic characteristics and prior infection between the included and excluded participants. Next, we calculated z-scores to detect outliers, defined as those with a z-score at least 3.5 SD greater than the standardized sample mean (43).

For the main analyses, we conducted an Exploratory Factor Analysis (EFA) of all 22 COVID-19 vaccination belief items to explore the underlying latent constructs and structure. We used maximum likelihood extraction with oblique rotation to allow factors to be correlated among each other (44). We set the Eigenvalue to one. We computed compound scores of all extracted factors averaging items with a factor loading of at least 0.30. Items with a loading of less than 0.30 and items with cross-loadings, i.e., at least 0.30 on more than one factor, were discarded. We tested internal consistency of all identified factors with Cronbach’s alpha, and retained for further analysis only those with an alpha of at least 0.70. Next, we ran zero-order correlations among the retained factors, the VAI, and vaccination intention as a final outcome measure. We conducted one-way ANOVA to compare vaccination intention, the VAI, and the retained factors among the four age groups (children, teenagers, adults, and older adults), and hierarchical regression analyses to test if sociodemographic characteristics, prior SARS-CoV-2 infection, VAI, and retained vaccination belief factors were associated with participants’ vaccination intention across all age groups.

## Results

### Sample Characteristics

The analytical sample included 1933 of the 2681 invited participants in the digital cohort study (72%). Response rates differed by age: 428 of 576 (74%) parents for their children; 222 of 394 (56%) teenagers; 842 of 1081 (78%) adults; and 441 of 630 (70%) older adults. Among respondents, 55% were female and mean age was 41 years (SD = 24). The modal level of highest educational attainment was apprenticeship/professional school (*n* = 668; 36%). The majority were Swiss (*n* = 1670; 87%), 236 (12%) European Union/EFTA, and 11 (0.6%) reported a nationality from another country. About half of the sample (51%) reported that their income was just the necessary to live on, and 50% worked either in the social/health care sector, with children <15 years, or clients or students ≥15 years. See Table 1 for sample characteristics.

**TABLE 1:** Sample characteristics, Corona Immunitas Ticino (Switzerland, Ticino, 2021).

	Respondents vaccination questionnaire(N = 1933)		Non-respondents (N = 748)		χ^2^-test/t-test^h^	*p*	General population in canton ticino^a^	
	*n*	%	*n*	%			*n*	%
Gender^a^					*2.145*	*0.143*		
Female	1061	54.9	386	51.7			180′350	51.3
Male	872	45.1	360	48.3			171′141	48.7
Age,^a^ ^,^ ^b^ (5+)	M = 40.9	SD = 23.8	M = 39.1	SD = 27.1	1.594	0.111	M = 47.16	SD = 22.48
5–13	428	22.1	148	19.8			29′000	8.5
14–19	222	11.5	172	23.0			20′343	5.9
20–64	842	43.6	239	32.0			208′833	61.0
65+	441	22.8	189	25.3			84′041	24.6
Highest educational attainment^c^ ^,^ ^d^					52.37	<0.001		
None/Obligatory school	70	3.7	77	18.0			85′951	24.3
Apprenticeship/professional school	668	35.5	240	33.8			89′842	25.4
High school	327	17.4	99	13.9			63′314	17.9
Higher professional training	135	7.2	59	8.3			47′397	13.4
University/university of applied sciences	680	36.2	236	33.2			67′205	19.0
Nationality^a^					22.98	<0.001		
Swiss	1670	87.1	596	80.9			2554′633	72.4
EU/EFTA	236	12.3	126	17.1			98′858	27.6
Other	11	0.6	15	2.0				
Number of household members^e^	M = 3.0	SD = 1.3	M = 3.1	SD = 1.4	1.88	0.060	—	—
1	221	11.6	79	10.8			65′301	40.0
2	549	28.8	208	28.5			48′960	30.0
3	364	19.1	135	18.5			23′633	14.4
4	556	29.1	179	24.6			19′028	11.6
5	172	9.0	100	13.7			5268	3.2
6+	47	2.5	28	3.8			1470	0.9
Perceived financial situation					24.47	<0.001		
More than the necessary to live	802	46.4	244	37.6			—	—
Just the necessary to live	885	51.2	369	56.9			—	—
Not enough to live	43	2.5	36	5.5			—	—
Job sector^f^					13.09	0.004		
Job in social/healthcare	95	12.8	46	20.6			92′829	62.2
Job with children <15 years	43	5.8	4	1.8				
Job with clients or students ≥15 years	233	31.5	66	29.6				
Other	369	49.9	107	48.0			56′525	37.8
Known prior COVID-19 infection (self)^g^					26.19	<0.001		
Positive	287	14.8	56	7.5			32′117	9.1
Negative/unknown	1646	85.2	692	92.5			—	—
Known prior infection (family member)					8.54	0.003		
Positive	267	13.8	137	18.3			—	—
Negative/unknown	1666	86.2	611	81.7			—	—

Note.

a Ticino population: Swiss Federal Statistical Office (FSO), 2020, N = 351′491 individuals.

b Age groups at time of random sampling by FSO, in May 2020.

c (Non-)respondents aged <20 assessed as “highest educational attainment among parents”.

d Ticino population: Structural Survey, FSO, Neuchatel,2017,N=353’709 individuals.

e Ticino population: Structural Survey, FSO, Neuchatel,2019,N=163’660 households.

f Ticino population: Structural Survey, FSO, Neuchatel, 2010, N = 149′354 individuals active in the labor market.

g Ticino population: https://www4.ti.ch/dss/dsp/covid19/home/; total number of positive tests as of 29.4.2021.

h When M(SD) is reported, independent samples t-test was applied.

Compared to non-respondents (*n* = 748), participants had a higher educational level, were in a better financial situation, were more likely to have a Swiss nationality, to work in contact with children, and were less likely to work in the social/healthcare sector. Approximately 15% had a prior infection and 14% were aware of a positive test result of a family member (Table 1).

Compared to official socio-demographics statistics from the Swiss Federal Statistical Office (FSO), older participants in Ticino were slightly underrepresented in the current sample, along with men, while non-Swiss nationals and those with a university degree were overrepresented (Table 1).

### Vaccination Intention by Age Group

**FIGURE 2:**
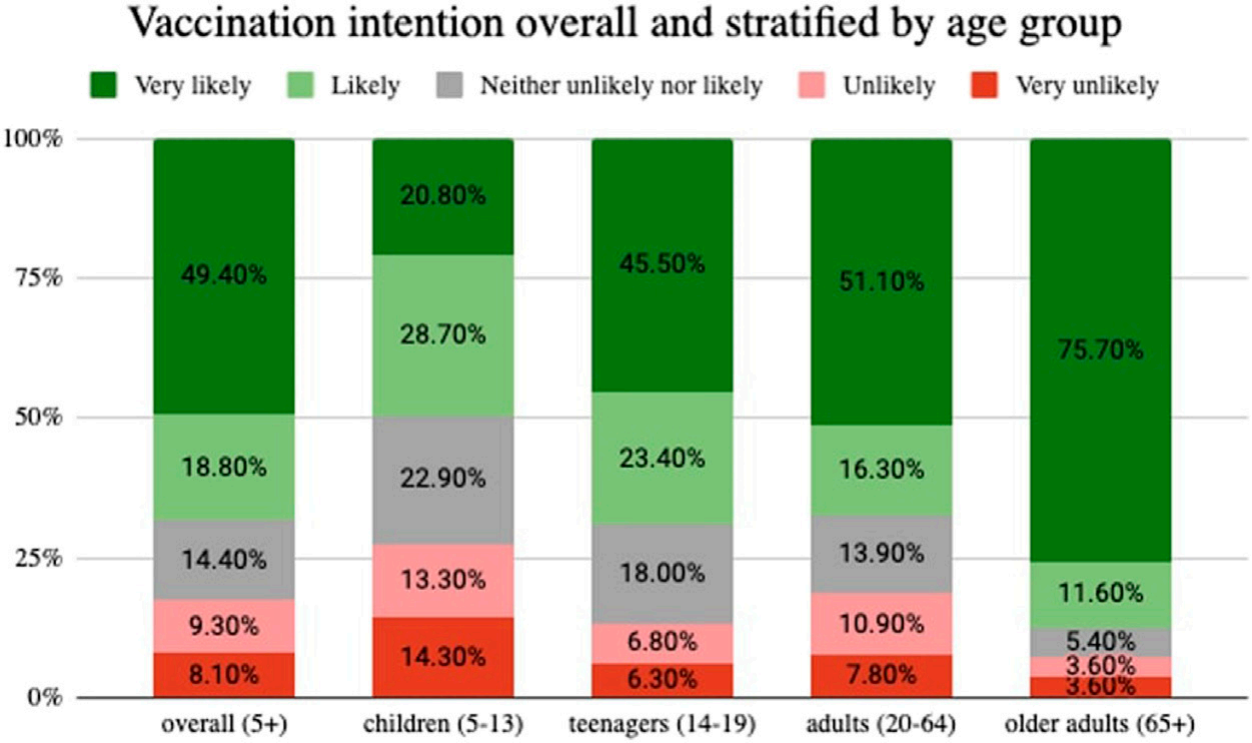
Vaccination intention overall and stratified by age group, Corona Immunitas Ticino (Switzerland, Ticino, 2021).

Two thirds (68%) of participants responded to be either “likely” or “very likely” to get vaccinated. Vaccination intention was higher among older adults (87%), followed by teenagers (69%), adults (67%), and parents who decide for their child (49.5%) (Figure 2).

One-way ANOVA results revealed significant differences in the mean intention score across age groups, except for teenagers and adults, who had similar scores (Table 2).

**TABLE 2:** Oneway ANOVA results for vaccination-related concepts stratified by age group (N = 1933), Corona Immunitas Ticino (Switzerland, Ticino, 2021).

	Parents of children (A)		Teenagers (B)		Adults (C)		Older adults (D)		*F* (3,1929)	Significant mean difference^a^
	M	SD	M	SD	M	SD	M	SD		
Vaccination intention	3.29	1.32	3.95	1.22	3.92	1.34	4.52	1.01	70.74***	AB, AC, AD, BD, CD
VAI	51.91	15.86	55.18	14.84	51.86	17.61	58.12	16.08	16.12***	AD, BC, CD
F1: WaitAndSee	3.95	0.97	3.21	1.08	2.98	1.25	2.52	1.19	116.35***	AB, AC, AD, BC, BD, CD
F2: ProtectAndMoveOn	4.51	0.64	4.30	0.66	4.23	0.74	4.40	0.74	15.98***	AB, AC, CD
F3: PreferenceForAlternatives	2.39	1.08	1.88	0.89	2.12	1.14	1.83	1.04	23.09***	AB, AC, AD, BC, CD
F5: ConfidenceInProtection	3.33	0.89	3.58	0.87	3.47	0.93	3.86	0.85	22.60***	AB, AC, AD, BD, CD

Note: Post hoc comparison based on Tukey HSD; *** *p* < 0.001.

a interpretation: AB, significant difference between group A (parents of children) and B (teenagers), the means presented in the respective columns indicate which of the two groups had a higher average score on the respective concept.

### Factor Analytic Results of Vaccination Beliefs

There were 211 outliers for six out of the 22 EFA items. Therefore, we ran the EFA twice (i.e., with and without outliers) and we gauged differences in the obtained pattern matrix. Because the number of factors did not differ in the two models, we included outliers in the final EFA model to capture possible deviant beliefs. We found five factors with Eigenvalues greater than one which, together, explained 58% of the common variance (Supplementary Table S2).

Three items loaded on Factor 1, which indicated a preference for more evidence on the vaccine and concerns about side effects. We thus labelled it “Wait and see.” Four items loaded on Factor 2, conveying a desire to contribute to the protection of one’s self, vulnerable people, and the society at large (“Protect and move on”). Four items loaded on Factor 3, indicating participants’ preference for natural alternatives and skepticism about its development (“Preference for alternatives”). Five items loaded on Factor 4 (“External and medical drivers”) potentially determining vaccination uptake. However, because the internal consistency across the five items was poor (Cronbach’s alpha = 0.504) we excluded Factor 4 from the final regression analysis. Four items loaded on the last factor regarding the belief that the COVID-19 vaccination protects from an infection and transmission of the virus (“Confidence in protection”). Supplementary Table S2 in the Supplement contains a summary of all factor analytic results. Descriptive statistics and zero-order correlations among all factors, vaccination intention, and the VAI are reported in Supplementary Table S3 in the Supplement.

We tested the robustness of our model running the abovementioned analyses twice (i.e., with and without imputed data for missing values). Because the composition and the descriptive results of the retained factors did not differ we used the data with imputations.

One-way ANOVA results showed that parents of children aged 5–13 were more prone to wait and see (Factor 1), to opt for alternatives to the COVID-19 vaccine for their child (once it is available) (Factor 3), and to question the protective value of the vaccine (Factor 5) relative to the other age groups (Table 2).

### Predictors of Vaccination Intention

Of the sociodemographics considered in Model 1 of our hierarchical regression analysis, age showed a significant and positive association with vaccination intention, an effect that endured through adjustment Table 3. In Model 2, we added known prior infection to the initial model (one’s own, or that of a family member). Knowing about one’s own past infection was not associated with vaccination intention but knowing about a prior infection of a family member significantly reduced intentions to vaccinate, another effect that endured through adjustment. In Model 3, we added acceptance of and trust in public authorities, measured in the VAI. The VAI was positively and significantly associated with intention, an association that remained significant also in the fully adjusted model (Model 4), where we added our identified factors of vaccination beliefs. The factors “Wait and See” and “Preferences for Alternatives” were significantly and negatively associated with vaccination intention, while beliefs reflecting “Confidence in Protection” were positively associated with intention to vaccinate. In the final model (Model 4), 71% of the overall variance in vaccination intention was explained by the included variables. The VAI (*β* = 0.309, *p* < 0.001) was associated with a 30% higher vaccination intention, similar to Factor 3 (“Preference for alternatives”; β = −0.294, *p* < 0.001), while Factor 5 (“Confidence in protection”; β = 0.182, *p* < 0.001), and Factor 1 (“Wait and see”; β = −0.143, *p* < 0.001) were associated with just below 20 and 15% higher vaccination intention, respectively.

**TABLE 3:** Hierarchical linear regression results predicting vaccination intention (N = 1933), Corona Immunitas Ticino (Switzerland, Ticino, 2021).

	Model 1				Model 2				Model 3				Model 4			
	B	SE	β	*p*	B	SE	β	*p*	B	SE	β	*p*	B	SE	β	*p*
Intercept	3.32	0.401			3.41	0.400			0.835	0.277			2.45	0.396		
Gender (female)	−0.242	0.140	—	0.085	−0.230	0.139	—	0.100	−**0.220**	**0.089**	—	**0.014**	−0.133	0.076	—	0.082
Age	**0.016**	**0.006**	**0.142**	**0.005**	**0.016**	**0.006**	**0.141**	**0.005**	**0.011**	**0.004**	**0.098**	**0.002**	**0.009**	**0.003**	**0.078**	**0.005**
Nationality (Swiss)^a^	−0.063	0.214	—	0.769	−0.054	0.213	—	0.801	−0.026	0.136	—	0.846	−0.017	0.116	—	0.880
Education (high)^b^	0.104	0.140	—	0.459	0.073	0.140	—	0.603	−0.046	0.089	—	0.606	−0.006	0.076	—	0.936
Financial situation (more than the necessary to live)^c^	0.066	0.140	—	0.640	0.066	0.139	—	0.636	−0.089	0.089	—	0.318	−0.128	0.076	—	0.092
Household size	−0.058	0.058	−0.050	0.313	−0.065	0.057	−0.057	0.254	−0.057	0.037	−0.050	0.118	−0.039	0.031	−0.034	0.210
Job in social/healthcare^d^	0.218	0.200	—	0.277	0.221	0.199	—	0.267	**0.268**	**0.127**	—	**0.035**	**0.219**	**0.109**	—	**0.045**
Job with children <15 years^e^	0.292	0.404	—	0.470	0.343	0.402	—	0.394	0.149	0.257	—	0.561	0.089	0.219	—	0.686
Known prior infection (self)					0.017	0.173	—	0.921	0.028	0.110	—	0.799	0.070	0.094	—	0.458
Known prior infection (family member)					−**0.495**	**0.187**	—	**0.008**	−**0.307**	**0.120**	—	**0.011**	−**0.263**	**0.102**	—	**0.010**
Vaccination Acceptance Index (VAI)									**0.056**	**0.002**	**0.757**	**<0.001**	**0.023**	**0.003**	**0.309**	**<0.001**
F1: WaitAndSee													−**0.152**	**0.040**	−**0.143**	**<0.001**
F2: ProtectAndMoveOn													0.086	0.055	0.049	0.117
F3: PreferenceForAlternatives													−**0.352**	**0.050**	−**0.294**	**<0.001**
F5: ConfidenceInProtection													**0.258**	**0.059**	**0.182**	**<0.001**
*F* statistics	*F* (8) = 1.85; *p* = 0.066				*F* (10) = 2.20; *p* = 0.017				*F* (11) = 58.28; *p* <0 .001				*F* (15) = 70.03; *p* <0 .001			
Adjusted *R* ^*2*^	*0.016*				*0.028*				*0.603*				*0.714*			

Note: B, unstandardized coefficient; SE, standard error; β, standardized coefficient; reference group:

a EU/EFTA, or Other.

b None/Obligatory school to Higher technical institute.

c Not enough or Just the necessary to live.

d Job with children <15 years, Job with clients or students ≥15 years, or Other.

e Job in social/health care, Job with clients or students ≥15 years, or Other.

Significant values.

The data met the assumptions of homogeneity of variance and linearity and the residuals were approximately normally distributed.

## Discussion

The aim of this study was to measure vaccination intention and significantly associated vaccine-related beliefs, in addition to socio-demographic factors, attitudes, trust, and prior infection with the SARS-CoV-2 virus in a representative sample of the population in Ticino, Switzerland.

We found that 68% of participants intended to get vaccinated. Of four factors capturing vaccine-related beliefs, three were significantly associated with vaccination intention, namely an inclination to wait for more evidence on safety and efficacy, a preference for alternative protection means, and confidence in the protective role of the vaccine. Moreover, participants wanting to wait for more evidence on the safety and efficacy of the vaccination had a significantly lower intention to get both themselves and their children vaccinated. This finding is in line with one survey of 3′000 Saudi residents, in which the majority of refusers reported they would accept the vaccine if additional studies confirmed safety and effectiveness (45). Evidence from the pre-COVID-19 era showed that lack of and need for more information and a vaccine’s novelty were the most frequently mentioned reasons for not vaccinating their children (46–48). In the present study, parents were significantly more inclined to “wait and see” more about the safety and efficacy of COVID-19 vaccines, and those with a preference for alternative protection means had lower intention to get vaccinated. Previous studies showed that vaccination rates increased when a mask-wearing policy was introduced, and that those who did not accept wearing a mask were more prone to accept vaccination (49). Finally, confidence in the protective role of the vaccine was a predictor of willingness to vaccination uptake both in ours and other studies (50, 51).

We used previously validated scales and conducted our study in a representative sample of the target population. Although previous studies differed by design, that 68% in our study intended to get vaccinated is consistent with similar estimates found in countries such as France, Germany, Canada, Singapore, Sweden, Nigeria (38), Turkey (52), Saudi Arabia (53), United States (27, 54) and United Kingdom (55), but is slightly lower than average acceptance estimates globally (26). Among parents of children 5–13, only 49.5% intend to vaccinate their child once a vaccine is available, while other studies conducted among parents/guardians found that vaccine acceptance was 65% (47) and 70% (55–57).

In our sample, vaccination intention increased with age, which is echoed by previous studies (26, 50, 51). Older adults may have greater concerns for their health compared to younger adults, and may be more prone to treatment and prevention. In Switzerland, older adults were offered the COVID-19 vaccination at the time of data collection. This may have contributed to a high salience of the topic and media coverage stressing the protective role of the vaccine in older populations. However, one study conducted in Saudi Arabia found that younger age was associated with higher acceptance of the vaccination (45). The different role of age in vaccination intention between countries warrants further investigations, and may be explained by geographic, contextual, and cultural differences.

Consistent with previous evidence, we found higher vaccination intentions among healthcare and social workers compared to the general population (55, 58). Yet, some studies found low acceptance rates among nurses (59, 60) and HCWs in general (61). To the best of our knowledge, there is no previous evidence on the relationship between one’s or a relative’s prior infection with the virus and COVID-19 vaccination intention. In this study, prior infection of one’s self was not associated with vaccination intention, but intention was lower among individuals who reported infection of a family member. Participants in our sample may consider infection-acquired and vaccine-acquired immunity distinctly different and may assume they are not mutually exclusive. Some evidence suggests that antibodies against the SARS-CoV-2 might wane over time, but it remains unknown how likely severe re-infection may occur (62). Nonetheless, the media was and still is covering the evidence on declining SARS-CoV-2 antibodies, often ambivalently (63). There may be a low perceived susceptibility to the disease in people with one or more relatives who had a prior infection because they may have presumed to have acquired natural immunity, irrespective of symptoms or testing. If the infected person experienced only mild symptoms their relatives may assume a similar course of the disease if infected, which may also contribute to diminishing the perceived need of personal protection provided by the vaccine.

Furthermore, our results on the association between trust in government and vaccination intention are in line with a recent survey in which respondents reporting higher levels of trust in information from government sources were more likely to accept a COVID-19 vaccine (26). Accordingly, individuals who are skeptical about vaccinations have also reported distrust in science and traditional medicine, with a preference for alternative remedies and prevention strategies (64, 65).

Participants who had lower vaccination intention also reported a preference to employ other protective means and wait until more information becomes available on the effectiveness and safety of vaccines. Our findings reiterate that reasons behind vaccine hesitancy are complex and include more than just an insufficient knowledge (66). To make an appropriate vaccination decision requires “the capacity to obtain, communicate, process, and understand basic health information and services” (67) (p.210), i.e., having an adequate health literacy level (68). This includes competence and skills in finding and discerning information from trustworthy sources, and to appraise, process, and use this information (69).

### Practice Implications

Our findings have several implications for practice. First, that a family member’s prior infection is negatively associated with one’s vaccination intention has an important implication for public health messaging. That having a family member with presumed antibodies against SARS-CoV-2 does not confer any protection on others in the household should be timely and effectively communicated to family members of individuals with a positive test. This is important also because new variants continue to emerge for which the antibodies acquired through exposure to the virus may become ineffective, while some types of vaccines can continuously adapt (70). The link between preference for waiting to know more about the vaccination and lower intention needs to be further unpacked, e.g., how much information is “enough” to take an informed decision? In addition, our results suggest that there are population subgroups who may believe that personal protective and social distancing measures confer greater protection than the vaccination. Communication efforts should address target audience’s specific beliefs and go beyond standard official statements and messages that vaccines are safe and effective (71, 72). This can be done, in part, by disseminating messages by locally trusted sources (73), and by maintaining a regular, transparent, bi-directional communication on the vaccination strategy with the public.

### Limitations

Some limitations are worth noting. First, issues of directionality because of the cross-sectional design. Vaccination intention may drive certain beliefs and preferences, such as the preference for alternative means of protection. Furthermore, vaccination intention was measured once, and scientific evidence, media coverage on the COVID-19 vaccine, and vaccination campaigns are rapidly changing, likely influencing vaccination intention. Second, potential social desirability bias cannot be excluded, but we intentionally retained outliers in our analyses to avoid a spurious masking of any polarized positions on the topic of the COVID-19 vaccination (74). Third, although response rate was high, self-selection bias is possible. Our sample was over-represented in terms of Swiss nationals, highly educated, and older participants. Participation in population-based research is generally lower among immigrants and higher among individuals of higher socio-economic status (75). The latter may have more trust in science, researchers, and medicine (76), and have altruistic motivations (77–79). However, the finding that more than one third of our participants reported to be either unlikely or unsure about getting vaccinated confirms the importance of learning about the vaccination-related beliefs of individuals from different sociodemographic backgrounds. Fourth, although hesitancy has been found to be higher for specific COVID-19 vaccines, e.g., in case they are manufactured in China (26), or in case of the Johnson and Johnson’s vaccine (80), we did not discriminate between vaccine types but asked to report on the COVID-19 vaccination, in general. At the time of data collection, only mRNA-based vaccines were available in Switzerland. Fifth, the recommendation of COVID-19 vaccines for teenagers 16 years or older, and the age-based priority strategy in the Swiss vaccination campaign at the time of data collection, potentially led to differences in the perceived relevance of the vaccination and the decision for or against vaccination uptake. Future studies on vaccination intention should, thus, be timely and consider the specific context of the eligibility of the study population. Last, the findings of our study may pertain to specific cultural and local features and should be generalized with caution to other regions in Switzerland, and to other countries (81, 82).
