# Supplementary material for: Why Vaccinate Against COVID-19? A Population-Based Survey in Switzerland
Source: Int J Public Health. 2022 Mar 23;67:1604226. doi: 10.3389/ijph.2022.1604226 (PMC8997237; doi:10.3389/ijph.2022.1604226)
Supplement: Supplementary file 1 [file DataSheet1.docx]

**Supplement Table 1.** Univariate descriptive statistics of vaccine-related items (N=1933), Corona Immunitas Ticino (Switzerland, Ticino, 2021)

| **Vaccination beliefs, attitudes, trust and intention** | **Range** | **M** | **SD** | **Skewness** | **Kurtosis** |
| --- | --- | --- | --- | --- | --- |
| 1. I prefer to wait before being vaccinated until more is known about how effective the vaccine is | 1-5 | 3.05 | 1.44 | -0.06 | -1.34 |
| 2. I prefer to wait before being vaccinated until more is known about the vaccine's safety | 1-5 | 3.14 | 1.47 | -0.15 | -1.37 |
| 3. I believe that the vaccination protects me against infection with the coronavirus | 1-5 | 3.77 | 1.11 | -0.71 | -0.22 |
| 4. I believe that the vaccination protects me against a severe course of coronavirus infection | 1-5 | 3.89 | 1.14 | -0.86 | -0.78 |
| 5. I believe that the vaccination protects against transmission of the coronavirus to others | 1-5 | 3.48 | 1.20 | -0.41 | -0.71 |
| 6. I am afraid of possible side effects | 1-5 | 3.16 | 1.32 | -0.10 | -1.13 |
| 7. I follow what my religious faith prescribes regarding this vaccination | 1-5 | 1.28 | 0.83 | 3.25 | 10.09 |
| 8. I prefer natural immunity against the coronavirus to vaccine-induced immunity | 1-5 | 2.27 | 1.36 | 0.71 | -0.74 |
| 9. I prefer natural or traditional remedies to the disease rather than being vaccinated | 1-5 | 1.80 | 1.17 | 1.42 | 1.00 |
| 10. I am afraid of injections | 1-5 | 1.88 | 1.32 | 1.27 | 0.25 |
| 11. I am concerned about getting infected if I go to a clinic where vaccinations are administered | 1-5 | 1.58 | 0.98 | 1.87 | 2.99 |
| 12. I would rather protect myself by other means (physical distancing, hand hygiene, wearing a mask) than be vaccinated | 1-5 | 2.19 | 1.26 | 0.80 | -0.42 |
| 13. I think that the vaccine will provide long-lasting immunity | 1-5 | 3.02 | 1.12 | -0.04 | -0.59 |
| 14. I want to protect myself | 1-5 | 4.22 | 1.04 | -1.34 | 1.14 |
| 15. I want to contribute to the protection of my community/society | 1-5 | 4.37 | 0.86 | -1.52 | 2.49 |
| 16. I want to contribute to the protection of someone I know who is vulnerable | 1-5 | 4.11 | 1.16 | -1.27 | 0.78 |
| 17. I want to get back to a normal life as fast as possible | 1-5 | 4.65 | 0.74 | -2.57 | 7.27 |
| 18. I prefer to let those who will benefit most have first access to the vaccine | 1-5 | 4.42 | 0.92 | -1.71 | 2.65 |
| 19. Medical reasons (e.g., allergies) prevent me from being vaccinated | 1-5 | 1.26 | 0.78 | 3.34 | 11.03 |
| 20. I base my vaccination decision on the results of my serological test | 1-5 | 1.83 | 1.20 | 1.29 | 0.54 |
| 21. The coronavirus vaccine has been developed too quickly | 1-5 | 2.83 | 1.36 | 0.16 | -1.12 |
| 22. I feel overwhelmed by information on the coronavirus vaccine | 1-5 | 2.65 | 1.34 | 0.27 | -1.07 |
| VAI 1: Thinking about vaccination in general, would you say you are personally…(“strongly against” to “strongly in favor”) | 0-90^1^ | 69.52 | 24.89 | -1.27 | 0.74 |
| VAI 2: I generally trust vaccine manufacturers or pharmaceutical companies | 0-80^2^ | 54.03 | 20.73 | -0.70 | 0.11 |
| VAI 3: I generally trust the Federal Office of Public Health (FOPH) | 0-80^2^ | 60.16 | 18.71 | -0.98 | 0.90 |
| VAI 4: I understand how vaccination helps my body fight infectious diseases | 0-80^2^ | 62.35 | 19.91 | -1.19 | 1.05 |
| VAI 5: I feel it is important that I get vaccinated | 0-80^2^ | 57.28 | 24.09 | -0.85 | -0.24 |
| VAI 6: Vaccination forms part of a healthy lifestyle | 0-80^2^ | 50.83 | 24.87 | -0.49 | -0.74 |
| INTENTION: Once the coronavirus vaccine is available to you (your child), how likely is it that you will decide to get (her) vaccinated? | 1-5 | 3.92 | 1.32 | -0.95 | -0.36 |

Note: ^1^converted from a 1-10 scale to a 0-100 scale, ^2^converted from 1-5 scale to a 0-100 scale

**Supplement Table 2.** Results from factor analysis on vaccination belief items (N=1933), Corona Immunitas Ticino (Switzerland, Ticino, 2021)

|  | Factor loading | | | | | | Cronbach’s Alpha |
| --- | --- | --- | --- | --- | --- | --- | --- |
|  | 1 | 2 | 3 | 4 | 5 |  | |
| Factor 1: WaitAndSee |  |  |  |  |  | .870 | |
| I prefer to wait before being vaccinated until more is known about the vaccine's safety | 1.00 |  |  |  |  |  | |
| I prefer to wait before being vaccinated until more is known about how effective the vaccine is | .912 |  |  |  |  |  | |
| I am afraid of possible side effects | .458 |  |  |  |  |  | |
| Factor 2: ProtectAndMoveOn |  |  |  |  |  | .745 | |
| I want to contribute to the protection of my community/society |  | .827 |  |  |  |  | |
| I want to contribute to the protection of someone I know who is vulnerable |  | .655 |  |  |  |  | |
| I want to protect myself |  | .627 |  |  |  |  | |
| I want to get back to a normal life as fast as possible |  | .409 |  |  |  |  | |
| Factor 3: PreferenceForAlternatives |  |  |  |  |  | .837 | |
| I prefer natural or traditional remedies to the disease rather than being vaccinated |  |  | .784 |  |  |  | |
| I prefer natural immunity against the coronavirus to vaccine-induced immunity |  |  | .754 |  |  |  | |
| I would rather protect myself by other means (physical distancing, hand hygiene, wearing a mask) than be vaccinated |  |  | .549 |  |  |  | |
| The coronavirus vaccine has been developed too quickly |  |  | .359 |  |  |  | |
| Factor 4: ExternalAndMedicalDrivers |  |  |  |  |  | .504 | |
| I am concerned about getting infected if I go to a clinic where vaccinations are administered |  |  |  | .546 |  |  | |
| Medical reasons (e.g., allergies) prevent me from being vaccinated |  |  |  | .512 |  |  | |
| I follow what my religious faith prescribes regarding this vaccination |  |  |  | .443 |  |  | |
| I base my vaccination decision on the results of my serological test |  |  |  | .386 |  |  | |
| I am afraid of injections |  |  |  | .328 |  |  | |
| Factor 5: ConfidenceInProtection |  |  |  |  |  | .815 | |
| I believe that the vaccination protects me against a severe course of coronavirus infection |  |  |  |  | -.878 |  | |
| I believe that the vaccination protects against transmission of the coronavirus to others |  |  |  |  | -.739 |  | |
| I believe that the vaccination protects against transmission of the coronavirus to others |  |  |  |  | -.586 |  | |
| I think that the vaccine will provide long-lasting immunity |  |  |  |  | -.427 |  | |
| *Explained variance (%)* | *28.8* | *11.0* | *8.5* | *5.1* | *4.6* |  | |

Note: The extraction method was maximum likelihood with oblique (direct oblimin) rotation. Kaiser-Meyer-Olkin measure of sampling adequacy = .873. Factor loadings below .30 are suppressed.

**Supplement Table 3.** Descriptive statistics and zero-order correlations of vaccination-related concepts (N=1933), Corona Immunitas Ticino (Switzerland, Ticino, 2021)

|  | M (SD) | 1 | 2 | 3 | 4 | 5 | 6 |
| --- | --- | --- | --- | --- | --- | --- | --- |
| 1 Vaccination intention | 3.9 (1.3) | 1 | .718** | -.612** | .364** | -.671** | .663** |
| 2 VAI | 53.7 (16.8) |  | 1 | -.512** | .466** | -.653** | .682** |
| 3 F1:  WaitAndSee | 3.1 (1.3) |  |  | 1 | -.185** | .587** | -.445** |
| 4 F2:  ProtectAndMoveOn | 4.3 (0.7) |  |  |  | 1 | -.298** | .449** |
| 5 F3: PreferenceForAlternatives | 2.1 (1.1) |  |  |  |  | 1 | -.544** |
| 6 F5: ConfidenceInProtection | 3.5 (0.9) |  |  |  |  |  | 1 |

Note: ** p < .01
